# 391. Small Towns, Big Cities: Rural and Urban Disparities Among Hospitalized Patients with COVID-19

**DOI:** 10.1093/ofid/ofab466.592

**Published:** 2021-12-04

**Authors:** Caroline Hamilton, Deepak Nag Ayyala, David Walsh, Christian-Jevon Bramwell, Christopher Walker, Rita Wilson Dib, Jessica Gosse, Amber F Ladak, Patricia Morissette, Arni S R Srinivasa Rao, Andrew Chao, Jose A Vazquez

**Affiliations:** 1 Augusta University/Medical College of Georgia, Augusta, Georgia; 2 Augusta University, Augusta, Georgia; 3 Medical College of Georgia @ Augusta University, Augusta, Georgia; 4 Medical College of Georgia, Augsuta, Georgia; 5 Medical College of Georgia at Augusta University, AUGUSTA, Georgia; 6 Augusta University Medical Center/Medical College of Georgia, Augusta, Georgia

## Abstract

**Background:**

More than half of all hospitals in the U.S. are rural hospitals. Frequently understaffed and resource limited, community hospitals serve a population that tends to be older and have less access to care with increased poverty and medical co-morbidities. There is a lack of data surrounding the impact of COVID-19 among rural minority communities. This study seeks to determine rural and urban disparities among hospitalized individuals with COVID-19.

**Methods:**

This is a descriptive, retrospective analysis of the first 155 adult patients admitted to a tertiary hospital with a positive COVID-19 nasopharyngeal PCR test. Augusta University Medical Center serves the surrounding rural and urban counties of the Central Savannah River Area. Rural and urban categories were determined using patient address and county census data. Demographics, comorbidities, admission data and 30-day outcomes were evaluated.

**Results:**

Of the patients studied, 62 (40%) were from a rural county and 93 (60%) were from an urban county. No difference was found when comparing the number of comorbidities of rural vs urban individuals; however, African Americans had significantly more comorbidities compared to other races (p-value 0.02). In a three-way comparison, race was not found to be significantly different among admission levels of care. Rural patients were more likely to require an escalation in the level of care within 24 hours of admission (p-value 0.02). Of the patients that were discharged or expired at day 30, there were no differences in total hospital length of stay or ICU length of stay between the rural and urban populations.

Baseline Characteristics of Hospitalized Patients with COVID-19

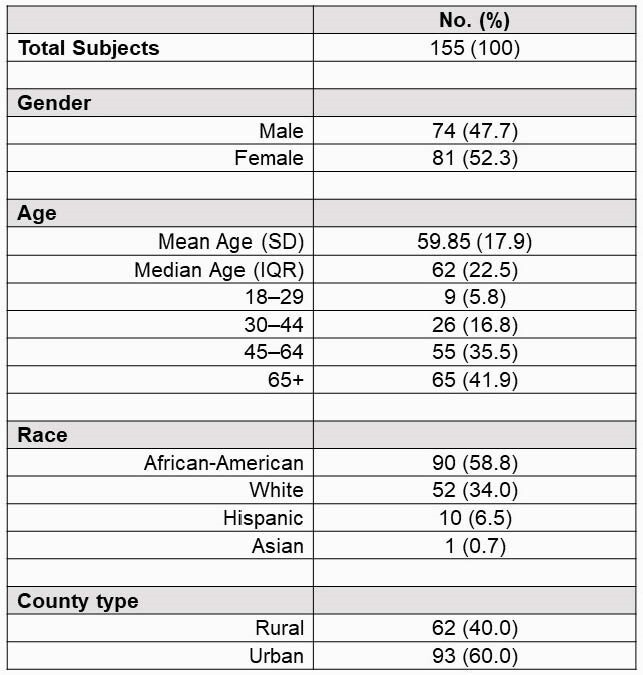

Day 30 Outcomes and Characteristics

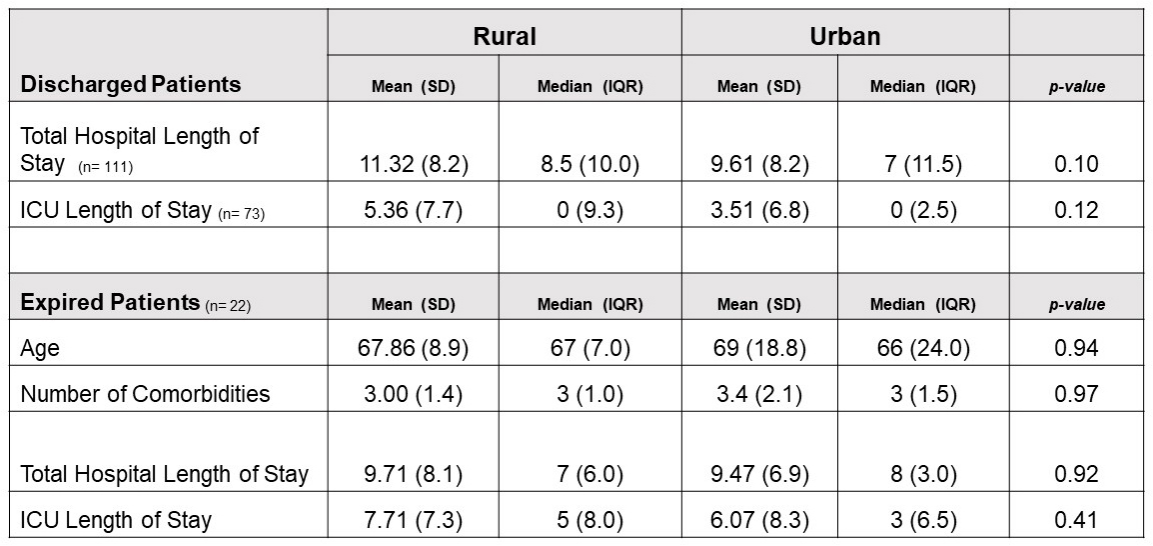

Level of Care at Time of Admission

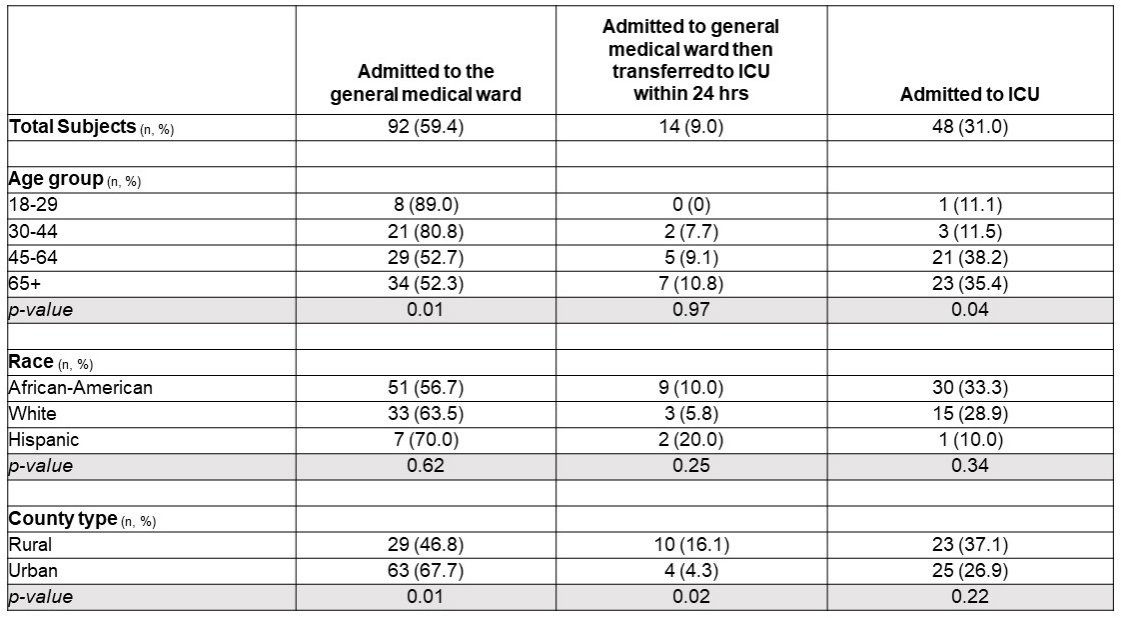

**Conclusion:**

This study suggests that patients in rural communities may be more critically ill or are at a higher risk of early decompensation at time of hospitalization compared to patients from urban communities. Nevertheless, both populations had similar lengths of stay and outcomes. Considering this data is from an academic medical center with a large referral area and standardized inpatient COVID-19 management, these findings may prompt further investigations into other disparate outcomes.

**Disclosures:**

**All Authors**: No reported disclosures

